# How Resiliency Affects Quality of Life Twenty-two Years Post-cardiac Transplant: a Case Report

**DOI:** 10.7759/cureus.1472

**Published:** 2017-07-14

**Authors:** Joslyn Vo, Davin Agustines

**Affiliations:** 1 Medicine, Scripps Mercy Hospital; 2 Semel Institute, UCLA School of Medicine

**Keywords:** resilience, cardiac transplantation, mental health

## Abstract

Heart transplantation has been shown to prolong survival significantly for people who have advanced heart disease. Even with improved prognosis, heart transplant recipients experience a lower overall quality of life compared to their healthy counterparts, which is correlated with high rates of depression and other psychiatric disorders. Our case report examines factors affecting the quality of life over a long period of time in a patient who received a heart transplant 22 years ago. This case is unusual because the patient overcame depression with just a minimal dosage of antidepressants due to her unwavering hope, resilience, and strong social support. This case serves as a reminder to always consider every aspect of a patient’s life, instead of just relying on high dose of medications, in order to improve their emotional and physical states and overall quality of life.

## Introduction

Heart transplantation (HTx) has resulted in the improved survival for people with advanced heart disease [1]. However, there are concerns regarding the quality of life in survivors, as heart transplantation can cause many psychological problems such as depression and anxiety [2]. Quality of life (QoL) is defined as an individual’s physical health, psychological states, level of independence, social relationships, and their relationship to salient features of their environment. A study of adolescent heart transplant patients found that medical regimen, the level of family conflict, and the parental quality of life predicted either physical or psychological functioning post the transplant. Particularly, fewer medication side-effects, lower level of family conflict, and better parent physical health were all associated with better adolescent physical functioning. Conversely, more medication side-effects correlated with poorer psychological health. In a multicenter study, adolescent HTx recipients reported excellent physical health; however, compared with healthy adolescents, they had significantly lower QoL scores for emotional, social, and school functioning [3]. Some of the predictors of better QoL include longer time post-transplant, ischemic heart failure, fewer barriers to health-promoting behaviors, higher levels of health competence, and a health-promoting lifestyle [4]. QoL was found to be negatively correlated with the prevalence of depression in HTx recipients. One study of 107 HTx adult recipients showed 14% of subjects had severe depression, and 31% had mild-to-moderate depression more than 10 years after HTx surgery [5]. Factors that predispose patients to post-transplant psychiatric disorders include pre-transplant psychiatric history, female gender, longer hospitalization, poorer physical health, and lower social supports from caregiver and family in the perioperative period [6]. Depression was also found to be associated with medication noncompliance, and depressed patients are three times less likely to adhere to medication treatment recommendation compared with non-depressed patients [7]. This is critical because heart transplant recipients need to be on multiple medications such as immunosuppressants. Thus, it is important to assess psychological health as a component of QoL in heart transplant patients.

Studies have shown that hope and resiliency have a significant effect on QoL. Lower levels of perceived hope are associated with higher levels of anxiety, depression, and hostility in female HTx recipients. Hope can independently predict moods and QoL [8]. Studies have also found a positive relationship between resiliency and a patient’s health. Resiliency has a strong inverse relationship with neuroticism but positive relationships with extraversion and conscientiousness [9]. Resiliency is a significant factor in positive adaptation after heart transplantation, which includes medical adherence, developing goals and life plans as well as achieving emotional balance [10]. Various studies have examined QoL in post-transplant patients, however, most have only observed this relationship at one point in time. In addition, previous studies recruited patients who were less than twenty years post-transplant. In this case report, we examine the effects of resiliency, hope, social support, and minimal antidepressants on QOL in a post-HTx patient over a longer period of time. We used a psychiatric evaluation and PHQ9 questionnaire at every visit to assess this patient’s psychological health.

## Case presentation

Our patient is a middle-aged Caucasian female who presented to our outpatient psychiatric clinic in April 2015 for the treatment of depression and anxiety. She had received a cardiac transplant 22 years prior, and since then has required two stents due to subsequent coronary arteriopathy. Unfortunately, she did not know which arteries had required stents, though she had gone for several years without any changes in cardiac function or worsening arteriopathy.

At the first visit, she presented with the Patient Health Questionnaire-9 (PHQ-9) of 19, which corresponds to moderately severe major depression. She reported significant feelings of depression and anxiety associated with her disease burden, feelings of physical inadequacy, and fears of social rejection from others’ reactions to her surgical history. She reported a significant history of both social rejection by peers, as well as employment discrimination, when her history of transplantation was revealed to others. As a result, she spent significant amounts of time and energy protecting her medical history from acquaintances, despite the need for an annual cardiac follow-up exam that would last one week, with a 2-3 week recovery periods.

Besides regular weekly psychotherapy, the patient was prescribed wellbutrin XL 150mg and zoloft 6.25mg daily, which is much lower than the Food and Drug Administration (FDA) approved dosage guideline of zoloft 50mg. Despite the low dosage of these antidepressants, over the next 8 months, the patient’s PHQ-9’s scores had progressively improved to 9, 5, and eventually 1.

## Discussion

What made her very unusual was the strength of her own initiative to continually build support networks, as well as seek out help even in the face of social and work rejection due to her transplant status. Even when in the midst of a severe depression, she actively sought out mental health help under her own initiative. Her resilience manifested in her staunch advocacy for herself, as well as her longstanding efforts to continually build social networks. Throughout her life, she had not endorsed any symptoms of hopelessness, despite periodic episodes of depression, as well as economic and social support setbacks. After her cardiac transplant, she focused on her schooling, and obtained a Master’s degree, despite the knowledge that median survival time post-cardiac transplant is 11 years. In spite of that, she remained hopeful and optimistic about her future. After her depression had resolved through the use of psychotherapy as well as minimal antidepressant dosages, she moved ahead and launched a new venture to become a small business owner. Throughout her life, she had very supportive parents and family that encouraged her to focus on the future. In so doing, they instilled in her the ability to adapt, as well as to continuously seek out other positive influences to encourage her in her pursuits to lead a normal life. She continues to work on building social networks outside her family; over the years, she has developed a moderately-sized group of friends and mentors that have chosen to support her emotional, intellectual, and economic pursuits, rather than emotionally distance themselves from her due to her different life trajectory. 

## Conclusions

This case report illustrates that not all patients with both comorbid medical illness and depression require the antidepressant dosages found in patients that have depression without comorbid medical illness. In addition, focusing treatment on improving an individual patient’s resilience, addressing external stressors in a patient’s life, and maximizing social support structure is critical towards improving long-term outcomes for patients. In this particular patient, her strong family support structure allowed her to grow emotionally, despite significant social rejection by acquaintances and co-workers due to her medical history. Growing up, she developed a strong drive to persevere, as well as hope. These are fundamental to developing emotional resilience. When we, as medical professionals, work with patients, we should also focus our time towards evaluating hopefulness, resilience, and coping skills in our patients. A better understanding of these aspects will allow us as professionals to work with patients in a holistic fashion by enabling us to treat the whole person, rather than just focusing our attention on the symptoms to be treated. This will ultimately lead to better patient outcomes, higher quality of life, and less use of medication in the patients that we treat.
